# Advances in the pathophysiology of adult-onset focal dystonias: recent neurophysiological and neuroimaging evidence

**DOI:** 10.12688/f1000research.21029.2

**Published:** 2020-03-26

**Authors:** Antonella Conte, Giovanni Defazio, Marcello Mascia, Daniele Belvisi, Patrizia Pantano, Alfredo Berardelli

**Affiliations:** 1 Department of Human Neurosciences, Sapienza, University of Rome, Rome, Italy; 2 IRCCS Neuromed, Pozzilli (IS), Italy; 3 Department of Medical Sciences and Public Health, Neurology Unit, University of Cagliari and AOU Cagliari, Monserrato, Cagliari, Italy

**Keywords:** dystonia, neurophysiology, neuroimaging, magnetic resonance; pathophysiology

## Abstract

Focal dystonia is a movement disorder characterized by involuntary muscle contractions that determine abnormal postures. The traditional hypothesis that the pathophysiology of focal dystonia entails a single structural dysfunction (i.e. basal ganglia) has recently come under scrutiny. The proposed network disorder model implies that focal dystonias arise from aberrant communication between various brain areas. Based on findings from animal studies, the role of the cerebellum has attracted increased interest in the last few years. Moreover, it has been increasingly reported that focal dystonias also include nonmotor disturbances, including sensory processing abnormalities, which have begun to attract attention. Current evidence from neurophysiological and neuroimaging investigations suggests that cerebellar involvement in the network and mechanisms underlying sensory abnormalities may have a role in determining the clinical heterogeneity of focal dystonias.

## Introduction

Dystonia is a disorder characterized by excessive and sustained muscle contractions that cause abnormal postures and involuntary movements that can be twisting, repetitive, or tremulous. It is often initiated or worsened by voluntary action and is associated with an overflow of muscle activity ^1, 2^.

Idiopathic adult-onset dystonia, the most common form of dystonia, has variable clinical expression, though it often has a focal onset such as blepharospasm (BSP), oromandibular dystonia, cervical dystonia (CD), laryngeal dystonia, or arm dystonia ^1, 3– 5^. In adulthood, the lower limb has rarely been observed as a site of dystonia ^6^.

The demographic and clinical characteristics of adult-onset focal dystonias are now well established. Dystonias in the craniocervical area are more common in women, whereas occupational limb cramps are more common in men ^3^. Adult-onset dystonia has a limited tendency to spread to adjacent body regions ^1, 3– 5^, which likely depends on the site and age of dystonia onset and on genetic factors ^7, 8^. Focal dystonias may be associated with rest/postural tremor in the head or upper limbs ^9, 10^.

In addition to motor signs, patients with adult-onset dystonia may also have a spectrum of nonmotor symptoms, including psychiatric manifestations (namely depression, anxiety, and obsessive compulsive traits) ^11^, mild disturbances in executive functions ^12^, and sensory symptoms ^13^. In patients with BSP, sensory symptoms may include a burning, gritty sensation in the eye, dry eye, and photophobia, which may develop months or years before BSP onset. In patients with CD, dystonic movements are often associated with neck pain that contributes significantly to patient disability and a low quality of life. The overall burden of nonmotor symptoms may vary in different patients with focal dystonia ^14^. All types of focal dystonia can be specifically relieved by sensory tricks, which are self-acquired maneuvers that transiently improve focal dystonia in a consistent proportion of patients ^15, 16^.

In an early paper, Marsden *et al*. ^17^ suggested that the basal ganglia play an important pathophysiological role in adult-onset focal dystonia. Indeed, early lesional studies showed that structural lesions in the basal ganglia determine dystonia (and not only focal forms) in contralateral body parts ^17– 20^. Since basal ganglia determine motor command in goal-directed motor learning (i.e. facilitate the desired motor output and concomitantly inhibit unnecessary motor output), basal ganglia dysfunction may conceivably determine abnormal motor command. Moreover, it was also observed that oscillatory activity of the globus pallidus internus (GPi) at a frequency of <12 Hz contributes to dystonic motor symptoms ^21– 23^ and that deep brain stimulation (DBS) of the GPi improved dystonia ^24– 26^.

Earlier neurophysiological studies demonstrated reduced inhibition at the level of the primary motor cortex, brainstem, and spinal cord ^1, 3, 27– 29^, abnormal plasticity mechanisms in the cortical motor areas, and abnormal sensory integration ^29– 34^. Likewise, magnetic resonance imaging (MRI) studies showed gray matter volume differences in several cortical/subcortical regions in patients with different focal dystonias. However, owing to the wide array of connections between the basal ganglia and several brain areas, these changes were generally interpreted as consequences of a primary basal ganglia dysfunction.

The assertation that focal dystonia is exclusively the result of basal ganglia dysfunction has recently been challenged by the remarkable clinical heterogeneity of the motor and nonmotor manifestations characterizing the different forms of adult-onset focal dystonias, as well as by lesion studies demonstrating that secondary focal dystonia is related to structural lesions in various sites in the nervous system, such as the basal ganglia, thalamus, and cerebellum ^35– 37^. It has therefore been proposed that dystonia may not be due to a lesion or an abnormal function of only one structure, namely the basal ganglia, but rather may be due to dysfunctional mechanisms in other brain areas, either concomitantly with or secondary to altered basal ganglia influence, that contribute to the pathophysiology of the condition. This may be particularly true for CD. Animal models of reversible dystonia induced by means of muscimol inactivation have suggested a dysfunction of a circuit including mesencephalic reticular formation neurons, cerebellum, tectum, and the basal ganglia, known to play a fundamental role in control of eye, head, and coordinated eye and head movements ^38– 44^. The dysfunction, however, may not be primary in these regions but rather reflect an abnormal activity originating in other structures providing feedback to the network such as the cerebellum. In this vein of thought, the role of the cerebellum in the pathophysiology of focal dystonias has attracted great interest in the last decade ^45, 46^.

In this paper, we provide a comprehensive overview of the new findings from recent neurophysiological and neuroimaging investigations regarding the pathophysiology of adult-onset dystonia and highlight the remaining knowledge gaps in the understanding of this condition.

## Recent evidence from neurophysiological investigations

In recent years, neurophysiological investigations have largely focused on the pathophysiological mechanisms linking the hypothesized basal ganglia dysfunction to the activity of distant sites and the mechanisms underlying sensory abnormalities in focal dystonias, specifically altered temporal discrimination of sensory stimuli and pain ( Table 1).

**Table 1.** Recent neurophysiological abnormalities in focal dystonias.

Neural structure	Function	Patients	Main findings
Cerebellum	Eye blink classical conditioning	CD with and without tremor	Altered only in patients with tremor
	Feedforward adaptation	CD with and without tremor	Altered only in patients with tremor
Sensory system	Somatosensory temporal discrimination threshold	CD	Increased values correlate with high-frequency oscillations and paired somatosensory evoked potentials inhibition
	Quantitative sensory testing	CD, upper limb dystonia, generalized dystonia	Reduced cold and hot detection threshold
	Laser evoked potentials	CD, BSP	Normal N2/P2 amplitude
	Conditioned pain modulation protocol	CD, BSP	Reduced conditioned pain modulation response as compared to patients with BSP and healthy subjects

BSP, blepharospasm; CD, cervical dystonia

Using scalp EEG recordings in dystonia patients implanted with DBS electrodes in the GPi and subthalamic nucleus, Miocinovic *et al*. ^47^ demonstrated that chronic DBS reduces exaggerated alpha oscillations and alpha band interhemispheric coherence in the motor cortex, thus confirming that clinical improvement with GPi-DBS reflects DBS-induced direct suppression of abnormal oscillatory activity in the motor cortex ^24, 48^.

Sedov *et al*. recently recorded single unit neural responses and local field potentials from the GPi in CD patients undergoing DBS surgery. Firing rate and discharge pattern of the GPi were asymmetric in patients with torticollis. Neuronal asymmetry correlated with the degree of involuntary head turning. Sedov *et al*. concluded that asymmetric pallidal activity results in asymmetric feedback to the mesencephalic neural integrator causing dysfunction in the network integrating eye and head coordinated movement ^49, 50^.

Studies using animal models of dystonia, however, also showed that cerebellar output alters basal ganglia activity and determines dystonic postures ^51– 53^. The observation that mutations in *THAP1* and *KMT2B* genes, highly expressed in the cerebellum, can induce generalized dystonia further supports the cerebellum’s role in the pathophysiology of dystonia ^54, 55^. In humans, cerebellar involvement in dystonia pathophysiology has recently been tested using eye blink classic conditioning (EBCC) and motor learning paradigms involving adaptation mechanisms. EBCC consists of a Pavlovian learning protocol integrated at the level of Purkinje cells and deep cerebellar nuclei. Some authors have shown that EBCC is impaired in patients with idiopathic focal hand dystonia and CD ^56^. Conversely, adaptation learning, which tests the predictive ability to adjust motor execution after a perturbation, has been found to be normal in patients with CD ^57, 58^. The contrasting findings obtained by these two neurophysiological tests tentatively exclude a global cerebellar dysfunction in dystonic patients. Since patients with dystonia, specifically those with CD, often have concomitant tremor, some authors have investigated whether the presence of tremor may be the clinical feature reflecting cerebellar involvement. Hence, in studying CD patients with and without tremor, Antelmi *et al*. ^59^ observed that patients with dystonic tremor showed a decreased number of conditioned responses in the EBCC paradigm as compared to healthy controls and dystonic patients without tremor. Similarly, when investigating anticipatory movement control during a bimanual task, Avanzino *et al*. ^58^ found that adaptation of anticipatory adjustment was altered in patients with CD and tremor but not in CD patients without tremor and healthy subjects. These observations therefore suggest that cerebellar dysfunction more likely determines tremor than dystonia. Nonetheless, since most neurophysiological investigations testing the cerebellar hypothesis were conducted in patients with CD, further investigations should also assess whether this conclusion also applies to other types of focal dystonia.

Earlier studies tested the sensory system in dystonic patients by assessing somatosensory-evoked potentials (SEPs), electrical potentials generated in sensory pathways at peripheral, spinal, subcortical, and cortical levels of the nervous system ^60^. In healthy subjects, the SEP amplitude obtained by stimulating two adjacent nerves simultaneously is smaller than that obtained by stimulating a single nerve due to inhibitory mechanisms ^61– 65^. In dystonic patients with upper limb involvement, some authors have reported impaired suppression of SEPs at the spinal, brainstem, and cortical levels after mixed stimulation of the median and ulnar nerves ^66, 67^, thus implying reduced inhibition at multiple levels of the sensory system. Supporting this hypothesis, several studies on patients with various forms of focal dystonia have reported increased temporal discrimination thresholds (STDTs), the interval needed to discriminate two consecutively applied stimuli ^30, 33, 68, 69^. Recently, Antelmi *et al*. ^70^ recorded the high-frequency potential oscillations (HFOs) related to SEPs in order to understand the mechanisms responsible for altered STDT in dystonia and found that patients with CD had a reduced area of the early component of HFOs and reduced paired SEP inhibition that correlated with increased STDT values. Since HFOs are generated by the activity of a population of 3b cortical inhibitory interneurons that receive thalamo-cortical inputs ^71^, the authors concluded that impaired temporal discrimination in dystonia arises from defective inhibitory mechanisms in the primary somatosensory cortex.

As regards pain, there is some evidence from quantitative sensory testing that the thermal detection threshold and pain sensitivity are abnormal in patients with dystonia ^72, 73^. Though an earlier investigation ^74^ showed, and a recent study confirmed ^65^, that nociceptive pathways are normal, as tested by laser-evoked potentials in patients with CD, Tinazzi *et al*. ^75^ recently investigated whether pain arises from dysfunction of regulatory pathways of nociceptive transmission. To this aim, the authors applied a protocol, termed the conditioned pain modulation protocol, to test descending inhibitory control on nociceptive neurotransmission. This protocol consists of delivering a painful conditioning stimulus alongside another experimentally induced painful test stimulus. The ratio of the laser evoked N2/P2 potential amplitude during the application of the heterotopic noxious conditioning stimulation, as compared to baseline, reflects the physiological reduction of the perceived conditioned stimulus. The authors found that patients with CD have a reduced conditioned pain modulation response as compared to patients with BSP and healthy subjects and concluded that the endogenous inhibitory pain system is primarily defective in CD. Although this abnormality was present regardless of the presence of pain in these patients, it is likely that this alteration makes patients with CD more susceptible to developing pain. In addition, the evidence that this response is normal in patients with BSP implies that the two types of focal dystonia may differ in their pathophysiological mechanisms ^75^.

## Recent evidence from neuroimaging techniques

The introduction of functional neuroimaging investigations has allowed investigators to view changes in the functional activity of various brain areas in dystonia ( Table 2). Changes in the blood oxygen level-dependent (BOLD) signal, measured while patients perform a task (task-based fMRI) or during resting conditions (resting state fMRI), allow the functional activity and connectivity of different brain areas to be evaluated ^76– 79^. Motor tasks such as hand movements, writing, playing instruments, and blinking are the most common tasks that have been used in dystonia. Sensory and motor task-related fMRI studies first showed an abnormal activation of the primary sensory and motor cortices, secondary motor cortex, basal ganglia, and the cerebellum that were consistent across different dystonia phenotypes ^80– 88^. Changes were detected while patients with dystonia performed tasks with and without dystonia induced by the movement itself and also when the tasks involved clinically unaffected body regions ^84, 85^. Altered sensory processing and abnormal somatotopic sensory organization in the basal ganglia and sensory cortex were also found, most consistently in the hand area of the primary somatosensory cortex ^80– 82^. Dysfunction was also detected during motor preparation and motor imagery ^83^.

**Table 2.** Recent neuroimaging abnormalities in focal dystonias.

MRI technique	Function/analysis	Patients	Main findings
Task-related fMRI	Head rotation	CD	Increased activation of the ipsilateral anterior cerebellum and sensorimotor cortex depending on the direction of head rotation
	Hand force task	CD	Increased activity of the cerebellum and decreased functional activity of the somatosensory cortex
	Visuospatial task	CD	Reduced activation of the cerebellum associated with a reduced connectivity of the cerebellum with basal ganglia and the motor cortex, and a reduced activation of temporal, premotor, and parietal associative areas
	Transient finger pressure	WC	Decreased activation of the sensorimotor network
	Visual looming stimuli	CD	Reduced superior collicular activation
Resting state fMRI	Independent component analysis	BSP	Abnormalities in sensory–motor network, frontoparietal network, salience network, default-mode network
	Intraregional brain activities and interregional functional connectivities	BSP	Abnormalities in both intraregional and interregional functional connectivities, abnormal functional connectivity between the right caudate and left striatum and right supplementary motor area
	Graph theoretical analysis	BSP, CD, WC, LD	Large-scale alteration of network architecture
Diffusion tensor MRI	FA, MD	BSP, CD, LD	Structural alterations distinguish between focal dystonia phenotypes

BSP, blepharospasm; CD, cervical dystonia; FA, fractional anisotropy; fMRI, functional magnetic resonance imaging; LD, laryngeal dystonia; MD, mean diffusivity; WC, writer’s cramp.

In the last three years, task-related fMRI studies have aimed to test the involvement of the cerebellum in focal dystonia and to identify functional correlates of altered sensory processing. In patients with CD, isometric head rotation in the direction of dystonic head rotation was associated with an increased activation of the ipsilateral anterior cerebellum, whereas isometric head rotation in the opposite direction was associated with increased sensorimotor cortex activity ^89^. In another study in CD patients performing a hand force task, symptom severity was associated with increased activity of the cerebellum and decreased functional activity of the somatosensory cortex ^90^. Using a visuospatial task, Filip *et al*. ^91^ found a reduced activation of the cerebellum associated with a reduced connectivity of the cerebellum with basal ganglia and the motor cortex, and a reduced activation of temporal, premotor, and parietal associative areas.

As regards sensory processing, temporal discrimination deficits corresponded to disrupted superior collicular activity during looming stimuli in patients with CD ^92^. In writer’s cramp, the sensory processing of stimulation sequences (transient finger pressure) before the execution of a motor task revealed widespread decreased activation of the sensorimotor network, suggesting defective sensory processing during motor planning in these patients ^93^.

In conclusion, recent evidence with fMRI has shown that the cerebellum is involved in altered connectivity and has identified functional correlates of altered sensory processing. However, since dystonic symptoms often worsen or are triggered by motor task execution, a relevant issue with task-related fMRI findings is that they are unable to determine whether functional abnormalities are causes or consequences of dystonic motor disturbances. Similarly, the cerebellar involvement in the abnormal connectivity can be either primary or compensatory. Studies in unaffected carriers of dystonia-related mutations and unaffected relatives of dystonic patients may help clarify this important issue.

Using resting-state fMRI with independent component analysis (ICA) ^94– 99^ in patients with BSP, Huang *et al*. ^100^ observed alterations in multiple neural networks including the sensory–motor network (decreased connectivity involving the bilateral primary sensorimotor cortex, supplementary motor area, right premotor cortex, bilateral precuneus, and left superior parietal cortex), the right frontoparietal network (decreased connections in the middle frontal gyrus, dorsal lateral prefrontal cortex, and inferior frontal gyrus), and the salience network (increased connectivity in the left superior frontal gyrus and middle frontal gyrus). Abnormalities in regions of the default mode network and sensory integration network have been reported in patients with BSP ^101^. Further confirmation of an abnormal functional connectivity between the right caudate and left striatum and right supplementary motor area correlating with BSP severity comes from the study by Ni *et al*. ^102^. In embouchure dystonia, changes in resting state connectivity were found in sensorimotor and auditory areas and in the cerebellum ^103^. Abnormalities in both intraregional brain activities and interregional functional connectivity were also described in patients with CD ^104^. Functional changes extensively involved both cortical and subcortical structures, and common alterations of the two measures were identified bilaterally in the postcentral gyrus as well as in the basal ganglia and thalamus. Overall, the above-cited findings with resting-state MRI have shown that in patients with focal dystonias altered connectivity in various brain networks is present independently from the execution of specific sensorimotor tasks, related or not to dystonia. Using both graph theoretical analysis ^79, 94, 105– 108^ and ICA, Battistella *et al*. ^109^ compared patients with task-specific (eight spasmodic dysphonia and seven writer’s cramp patients) and non-task-specific dystonia (nine CD and nine BSP patients) and found that every patient exhibited unusually expanded or minimized neural communities. In addition, patients with task-specific dystonia had substantial connectivity alterations in the primary sensorimotor and inferior cortices and abnormally formed hubs in the insula and superior temporal cortex, as compared to patients without task-specific dystonia ^109^. Results from graft theory analysis, therefore, suggest a large-scale alteration of network architecture in focal dystonia, with distinguishing features between task-specific and non-task-specific dystonia.

Earlier diffusion tensor imaging (DTI) ^79, 110– 112^ studies showed microstructural alterations in the fiber tracts connecting the brainstem nuclei, basal ganglia, thalamus, cerebellum, motor cortex, and sensory cortex, and in the white matter (WM) of limbic, occipital, and prefrontal cortices in different forms of focal dystonia ^79, 113, 114^. In recent years, Berman *et al*. ^115^ used DTI in patients with BSP and CD to show that there are focal alterations in various brain structures that are specific to the two forms of focal dystonia (i.e. GPi, subthalamic, and red nuclei in BSP versus the caudate nucleus and cerebellum in CD). Consistent with the hypothesis that specific focal alterations may distinguish between focal dystonia phenotypes, Bianchi *et al*. ^116^ observed that spasmodic dysphonia phenotypes may be distinguished on the basis of focal structural abnormalities in the areas of motor control of speech production and auditory–motor integration, whereas spasmodic dysphonia genotypes were associated with structural changes in higher-order extra-Sylvian regions and their connecting pathways. Similarly, differences in structural integrity have been reported by Kirke *et al*. ^117^ in patients with spasmodic dysphonia, both with and without tremor. Compared to patients with spasmodic dysphonia without tremor, patients with spasmodic dysphonia and tremor exhibited a greater extent of WM changes in the right posterior limb of the internal capsule at the junction of the corticospinal/corticopontine tracts and superior thalamic radiation ^103^.

Finally, Corp *et al*. ^37^ used an MRI technique termed “lesion network mapping” from connectome data of a large cohort of healthy subjects to test whether lesions causing CD belong to a common brain network. However, the authors found that patients with CD had heterogeneous lesion sites (basal ganglia, brainstem, and cerebellum) that all belonged to a single functionally connected brain network.

## Conclusion

For a long time, the pathophysiology of adult-onset focal dystonia has been thought to involve, uniquely, a basal ganglia disturbance. However, accumulating evidence in recent years now points to the anatomical and functional involvement of several brain regions. Recent findings have demonstrated an association between acquired focal dystonia and lesions in various brain regions, including the cortex, the basal ganglia, thalamus, brainstem, and cerebellum. Convincing evidence from both neurophysiological and neuroimaging investigations supports the hypothesis that the cerebellum intervenes in the pathophysiology of dystonia. However, although it is widely accepted that abnormal cerebellar output may determine tremor in dystonia, it is still unclear whether the cerebellum is the primary node where aberrant communication arises. Moreover, it is also unclear whether the cerebellum plays a substantial role in all forms of focal dystonia or only in CD.

The neurophysiological reports of impaired sensory and motor inhibition at multiple levels of the central nervous system (contributing to altered tactile temporal discrimination as well as to motor manifestations) may well reflect not only a direct basal ganglia disturbance but also disturbances in the activity of basal ganglia-thalamo-cortical and cerebello-thalamo-cortical projections. Pain in CD patients, likely due to impaired descending regulatory mechanisms of nociceptive transmission, implies an additional dysfunctional network in this form of focal dystonia. In summary, recent neurophysiological and neuroimaging studies demonstrate that focal dystonias involve disordered communication among several brain networks, in which basal ganglia and, possibly, the cerebellum act as entraining structures. Although different forms of focal dystonia all share alterations in neural structures belonging to the basal ganglia-thalamo-sensorimotor cortical network, specific clinical dystonic features may also emerge because of characteristic neural signatures in specific networks.

Finally, increased attention is now directed towards the remarkable clinical heterogeneity of motor manifestations and the variable occurrence of sensory, psychiatric, and executive function disturbances. The heterogeneous clinical features together with the neurophysiological and neuroimaging advances support the trend towards “splitting” focal dystonias, insofar as, taken to the extreme, each focal dystonia is unique to each patient.

## Abbreviations

BSP, blepharospasm; CD, cervical dystonia; DBS, deep brain stimulation; DTI, diffusion tensor imaging; EBCC, eye blink classic conditioning; GPi, globus pallidus internus; HFOs, high-frequency potential oscillations; ICA, independent component analysis; MRI, magnetic resonance imaging; SEPs, somatosensory-evoked potentials; STDT, increased temporal discrimination threshold; WM, white matter.
